# Expanding Early Psychosis Care across a Large and Diverse State: Implementation Lessons Learned from Administrative Data and Clinical Team Leads in Texas

**DOI:** 10.1007/s10488-023-01285-8

**Published:** 2023-08-02

**Authors:** Deborah A. Cohen, Vanessa V. Klodnick, Samantha J. Reznik, Molly A. Lopez

**Affiliations:** 1 Dell Medical School Department of Psychiatry and Behavioral Sciences, The University of Texas at Austin, 1601 Trinity St., Bldg, B., Austin, TX 78712 USA; 2 Steve Hicks School of Social Work, The University of Texas at Austin, 1925 San Jacinto Boulevard, Austin, TX 78712 USA; 3 Texas Institute for Excellence in Mental Health, The University of Texas at Austin, 1925 San Jacinto Boulevard, Austin, TX 78712 USA

**Keywords:** Coordinated specialty care, First episode psychosis, Evidence-based practices, Implementation science

## Abstract

The U.S. is facing an unprecedented youth mental health crisis. Translating the findings from mental health intervention trials into large scale, accessible community-based services poses substantial challenges. Examination of state actions as a result of research-informed federal policy to improve youth access to quality mental healthcare is necessary. This mixed-methods study examines the implementation of evidence-informed multidisciplinary *coordinated specialty care (CSC)* for first-episode psychosis (FEP) services across Texas. The study explores CSC service model components, site location and participant characteristics, and implementation barriers. This cross-sectional study analyzes State of Texas public mental health administrative data from 2015 to 2020, including CSC site (n = 23) characteristics and CSC participant (n = 1682) demographics. Texas CSC site contracts were compared to OnTrackNY, a leading CSC model in the U.S. for CSC service element comparison. In-depth interviews with CSC Team Leads (n = 22) were analyzed to further understand CSC service elements and implementation barriers using qualitative content analysis. CSC was implemented across three waves in 2015, 2017, and 2019—serving 1682 participants and families. CSC sites were located in adult mental health programs; approximately one third of CSC participants were under 18 years. CSC implementation challenges reported by Team Leads included: staff role clarification, collaboration and turnover, community outreach and referrals, child and adult service billing issues, and adolescent and family engagement. Study findings have implications for large state-wide evidence-based practice implementation in transition-to-adulthood community mental health.

The U.S. is experiencing a youth public mental health crisis (U.S. Surgeon General, 2021). There are several federal initiatives and new policies aimed at improving youth mental health care access and quality. For the US to be successful in building an effective youth mental healthcare continuum across the country, we need to better understand how states are interpreting federal policy and implementing evidence-based practice models that span both child and adult mental health systems. In the U.S., child and adult mental health service systems are largely separate, yet there is increasing recognition of the unique needs of the transition-to-adulthood population (Sabella, Davis, & Munson, 2020). The relatively swift adoption and implementation of coordinated specialty care (CSC) for first-episode psychosis in the U.S. provides a unique opportunity to better understand state efforts to meet transition-age youth mental health needs.

## Psychosis and Societal Disparities

Schizophrenia spectrum conditions are heavily stigmatized and associated with lifelong disparities, including social isolation or exclusion, poverty, homelessness, psychiatric hospitalizations, and disability benefit use (Charlson et al., 2018; Chen et al., 2021; Chong et al., 2016; Folsom et al., 2005; Harvey et al., 2012; Luciano & Meara, 2014; Read, 2010; Rosenheck et al., 2017; Velthorst et al., 2017; Wickham et al., 2014). In the last 20 years, schizophrenia treatment research has shifted to early detection and treatment in youth and young adults (Bird et al., 2010; Craig et al., 2004; Frawley et al., 2021; Kane et al., 2016; Petersen et al., 2005) leading to NIH investment in the *Recovery After an Initial Schizophrenia Episode (RA1SE)* studies that created the US Coordinated Specialty (CSC) model (Alcover et al., 2019; Dixon et al., 2015; Kane et al., 2016; Nuttall et al., 2019; Robinson et al., 2019; Rosenheck et al., 2016). CSC aims to intervene as early as possible with a mix of psychiatric, clinical, vocational, and peer supports (Dixon et al., 2015; Heinssen et al., 2014; Mueser et al., 2015) to decrease duration of untreated psychosis, which is associated with poorer long-term outcomes (Bertolote & McGorry, 2005; Birchwood & Fiorillo, 2000; Boonstra et al., 2012; Bottlender et al., 2003; Howes et al., 2021; Marshall et al., 2005).

### Translation of Science into Practice in Community Mental Health

Given the positive findings found in the *RA1SE* studies (Alcover et al., 2019; Dixon et al., 2015; Kane et al., 2016; Nuttall et al., 2019; Robinson et al., 2019; Rosenheck et al., 2016), in 2014, SAMSHA directed states to allocate 5% of their Mental Health Block Grant (MHBG) funding to pilot first-episode psychosis programs. This funding increased to 10% in 2016 and maintained at 10% annually since (Rosenblatt & Goldman, 2019). CSC expanded from approximately 12 sites in 2010 to 162 sites by 2016 (Pollard & Hoge, 2017). As of 2019, 49 states had at least one FEP treatment site, with over 260 sites across the U.S. (NIMH, 2022). The rapid expansion of CSC from research trial to real-world implementation has challenged research, policy, and practice communities to ensure programs can replicate the positive results found in efficacy trials and address the many issues that arise with complex interventions and systems. CSC differs from other mental health services in that it serves adolescents and young adults that typically would get care from either a child provider (up until age 18, with some grant-driving exceptions in Texas) or an adult provider (age 18 and older) if diagnostic eligibility criteria are met (Cohen et al., 2020a, 2020b). U.S. child and adult providers have different (and sometimes conflicting) service principles, policies and practices (Cohen et al., 2020a, 2020b; Davis et al., 2006).

Despite the unique features of CSC and its aim to serve both youth and young adults, comparatively less research has specifically unpacked implementation barriers. Powell et al. (2021) reviewed CSC literature and identified several implementation barriers, including: stigma, cultural competence, disengagement, measurement and evaluation, workforce development, implementation in rural areas, and financial sustainability. Other researchers have identified political and social factor barriers, such as having support and relationships with policymakers, community partners, and integrating user narratives to advocate for CSC services (Csillag, 2018) as well as funding and structural barriers (Bao et al., 2021; Dixon, 2017; Smith et. al, 2019). Furthermore, limited research has included provider perspectives. Stokes et al. (2022) sought provider perspectives from five US CSC sites, highlighting provider/staff level barriers (e.g., service delivery, demands on time, training/consultation) and organizational-level barriers (e.g., referrals, organizational climate, hiring and retaining providers, and financial sustainability). Researchers have detailed CSC implementation in New York State and California (Niendam et. al, 2019). The present study aims to detail CSC implementation process from administrative data and CSC providers within the unique Texas mental healthcare landscape, illuminating several possible barriers for other US states aiming to implement multidisciplinary community mental health services for transition-age youth.

Mixed-methods are critical for increasing our understanding of evidence-based practice implementation barriers across multiple sites. States and providers collect incredible amounts of data regularly that goes unused, and has the potential to inform evidence-based practice implementation. Fidelity scales are the gold standard for assessing evidence-based practice model adherence. In CSC, there are multiple fidelity scales, and states across the US were mandated to design their own evidence-informed approach to CSC. Some states adopted specific CSC models (e.g., OnTrackNY, NAVIGATE), while other states drew from these models and constructed their own models. This makes assessing fidelity to CSC incredibly complicated in the US. Texas has assessed fidelity to CSC using an OnTrackNY-informed scale, see Lopez et al. (2021). This study specifically aims to use administrative data, state contracts, and CSC Team Leveraging multiple data sources (e.g., administrative data, state contracts, site interviews) can help us to better understand how CSC implementation occurs in real world settings, as well as, perceived barriers to CSC implementation that could impact fidelity.

### Study Purpose

This study aims to (1) describe locations, demographics, and service delivery models in Texas based on administrative data and (2) identify Team Leads’ perceived implementation barriers in Texas’s roll out of evidence-informed community-based mental health care targeting both child and adult populations simultaneously. Research questions include: (1) Where are CSC sites located across Texas (geographic location; child vs. adult service site); (2) What are CSC participant demographics by site in this state; (3) What elements does the Texas state contract outline as part of the CSC model and how do these compare to a nationally implemented CSC Model (i.e., OnTrackNY) and (4) What are CSC implementation challenges reported by CSC Team Leads?

## Methods

The University of Texas at Austin Institutional Review Board and Texas Health and Human Services Commission Institutional Review Board reviewed and approved this project as not constituting human subjects research. This study uses three data sources: (1) CSC site program managers or team supervisor (referred to as “Team Leads” below and throughout the paper) interviews, (2) public mental health administrative data, and (3) Texas CSC provider contracts and OnTrackNY manual comparison. Each of these three data sources is described in further detail below.

### Setting

Texas is the second most populous and geographically large U.S. state, and is diverse in geography, race, ethnicity, language, and immigration status. Texas’ public mental health system is geographically segmented into 39 separate centers that cover singular counties in the large metropolitan areas (e.g., Dallas, Houston) or cover up to 23 rural counties (i.e., West Texas). The public mental health providers included in this study (23 of the 39) cover 94 of the 254 counties, representing over 75% of the population of the state. Of the sites included in the interviews, seven served one county, seven programs only served their most populous county (if they serve multiple counties), and nine served their entire catchment area across multiple counties. Since these interviews the sites serving only selected areas of their catchment have expanded coverage. Due to Texas’ vast geographic size and variability, the state mental health authority prefers to pilot new approaches in one or more urban areas, and then slowly expand across the state, learning lessons along the way about adaptions needed to meet the diverse needs of the state. No Texas sites were included in the original RA1SE-ETP study, and original RA1SE study CSC rural sites (Kane et al., 2016) did not meet the USDA definition of a frontier and remote area like several rural counties in Texas. 81.1% of Texas counties (206 of 254) were designated as either full or partial mental health shortage areas in 2015 (Hogg Foundation, 2020).

### Texas CSC Expansion Story

Most states used MHBG funds to support CSC implementation across a variety of settings, including academic medical and community mental health centers. Similar to a few other states (e.g., Illinois), Texas provided MHBG funding to implement CSC only through its 39 public mental health centers. In 2015, the Texas Health and Human Services Commission (HHSC) piloted CSC in two urban sites, one in Houston and one in Dallas (i.e., CSC Cohort 1). In 2016, eight more CSC sites were added by the state mental health authority (i.e., CSC Cohort 2). In 2019, Texas expanded to a total of 28 CSC teams within 24 community mental health centers (i.e., CSC Cohort 3). At the time of this publication, Texas had begun expansion for CSC Cohort 4, which includes six new centers, and increased additional funding to the most populous areas to allow them to expand the number of teams within their agency for a total of 42 teams at 29 community mental health centers. One of the Cohort 2 sites closed their CSC team in 2022.

During Cohort 1 Texas CSC implementation, Texas HHSC partnered with OnTrackNY (Bello et al., 2017) for training and implementation support at the two pilot sites. In subsequent CSC expansions, Texas HHSC provided sites with funding to support training, but did not prescribe training on a specific CSC model or training organization. Rather, Texas HHSC encouraged a CSC site peer-to-peer mentorship model in which older teams supported newer sites. As new CSC sites joined the Texas CSC expansion, Texas HHSC encouraged sites to consult with previously established sites to identify strategies to support implementation. Houston and Dallas CSC programs encouraged the second cohort to utilize OnTrackNY training, and the third cohort received the same suggestion. This site peer-to-peer support model led to the State of Texas *unofficially* adopting the OnTrackNY CSC model (Bello et al., 2017). During initial implementation of each CSC program, all sites reported attending in-person OnTrackNY training, while a few were currently (n = 5) using OnTrackNY online training for on-going training. All sites participated in monthly OnTrackNY consultation calls during the first year of CSC implementation.

### Data Source 1: CSC Team Lead Interviews

At the time interviews took place (July 2020), there were 23 CSC sites with clients actively serving individuals across Texas. Four sites had two CSC teams serving their large catchment area that reported up to a shared supervisor over both teams. The rest of the organizations had one CSC team. Texas HHSC provided CSC team lead e-mail addresses to the research team. The research team emailed Team Leads about the opportunity to participate in a one-hour interview. Sixteen Team Leads signed up for the interview after one invitation, five Team Leads signed up after two invitations, and one declined participation. Twenty-two Team Leads participated. We describe the qualitative process below using the SRQR checklist (O’Brien et al., 2014).

Before the interview, Team Leads verbally consented, and the interview was audio recorded. A bachelors-level research assistant interviewed participants via phone. He was trained in interviewing best-practices and supervised by a PhD level mixed-methods intervention researcher. The research assistant did not have relationships with any of the team leads, but had participated in meetings, trainings, and conferences with some of the team leads. The research assistant used a structured interview guide with questions that included: *“What roles does your CSC team have? What training has your team received? What challenges has your team faced in implementing CSC?”* Interviews lasted between 45 and 60 min.

The four-person research team transcribed each interview verbatim, engaged in individual open-coding to identify initial ideas and perspectives that appeared frequently and were salient. The team met and discussed initial thoughts and impressions as a group, co-identifying several implementation barriers. The team then independently applied qualitative content analysis (Schreier, 2013) to identify implementation barriers and related factors using the following analytic questions: *“Per Team Leads, how and why is this barrier occurring or not occurring? Under what conditions do Team Leads find this barrier occurring or not occurring?”* Themes were directly derived from the data. After reviewing 2–3 interview transcripts independently, the research team met to discuss emergent thematic patterns and unique experiences/perspectives, writing memos, and documenting all code construction. This process was repeated until all 22 CSC site Team Lead interviews were coded, and the themes matrix was updated. The team then reviewed the themes matrix together to develop and organize the results section of this paper.

### Data Source 2: Public Mental Health Administrative Data

All 39 public mental health providers in Texas submitted data on individuals served through all state-funded mental health programs. The dataset includes demographics of all individuals served arranged by service agency. The state data includes: client’s primary ICD-10/DSM-V diagnoses, service utilization data for each individual service rendered, individual item scores from the state’s mandated assessment: Child Needs and Strengths Assessment/Adult Needs and Strengths Assessment (Lyons et al., 1999; Lyons & Walton, 2013), and the level of care recommended and authorized. Public mental health providers in Texas use a state-mandated level of care (LOC) system to manage care within the resource-limited safety net structure. Following a similar model found in many U.S. states’ public mental health agencies, all children and adults entering mental health services obtain a diagnostic evaluation and complete the state-mandated assessment to determine functional strengths and needs. After completing the diagnostic evaluation and the state-mandated assessment, a level of care is recommended using an algorithm to identify the intensity of services to be provided. For specialty programs such as CSC, the individual can be directed to the “Early Onset” level of care. The research team used developed a matrix of team characteristics including details on team roles, counties served, rurality, paired with CSC implementation barriers from the team lead qualitative analysis.

### Data Source 3: Comparison of Texas CSC Provider Contract and OnTrackNY manual

Finally, to explore model implementation standards, the Texas CSC Provider Contract was compared to the OnTrackNY manual to assess for interpretation differences between the model that teams were trained on and requirements outlined in the provider contract. To ensure consistency across sites, Texas HHSC uses contracts to outline required services for special projects, such as CSC. The contracts outline minimum standards expected of each site, data reporting requirements, and allow for flexibility in staffing depending upon the rurality of the site. The research team examined similarities and differences between the Texas CSC Provider Contract and OnTrackNY manuals.

## Results

### CSC Across State of Texas

At the time of data collection (July 2020), CSC funding covered one third of Texas counties (74 of 254) through 24 of the 39 public mental health providers. However, one site had delayed implementation, leading to only 23 sites being included in this study. There was extensive variability in size and geographic service coverage within the agencies housing CSC programs. For example, one agency serves *Harris County,* a large populous county that includes the city of Houston; while another agency serves 23 rural counties in West Texas. Based on Team Lead interviews, 17 CSC sites serve their entire catchment area, and six serve one or more selected counties, generally representing the most populous areas of their catchment** (**see Table 1).

**Table 1:** Description of coordinated specialty care program sites in Texas

Site	Program Start	Location Type	No. Counties Served
#1	Cohort 3	Rural	5/5
#2	Cohort 2	Suburban/Rural	7/8
#3	Cohort 3	Rural	4/4
#4	Cohort 2	Rural	12/12
#5	Cohort 2	Urban	1/1
#6	Cohort 3	Rural	5/5
#7	Cohort 3	Rural	9/9
#8	Cohort 3	Rural	6/9
#9	Cohort 3	Suburban	1/1
#10	Cohort 2	Urban/ Border	1/1
#11	Cohort 2	Urban	1/1
#12	Cohort 3	Suburban	1/1
#13	Cohort 1	Urban	1/1
#14	Cohort 2	Urban	1/1
#15	Cohort 3	Rural	2/6
#16	Cohort 3	Rural	4/4
#17	Cohort 3	Rural	2/6
#18	Cohort 2	Rural	2/21
#19	Cohort 3	Rural	3/3
#20	Cohort 1	Urban	1/1
#21	Cohort 3	Rural	3/3
#22	Cohort 2	Urban/Rural/Border	3/3
#23	Cohort 3	Rural	5/23

^*^Cohort 1 sites began in 2015. Cohort 2 sites began in 2017. Cohort 3 sites began in 2019

### CSC Participant Demographics

Between 2015 and 2020, 1682 individuals participated in CSC programming in Texas. There was a similar ratio of children to adults across sites in Cohorts 2 and 3, but Cohort 1 sites enrolled fewer children to adults. Children represented 30% of CSC participants across Texas. Table 2 illustrates CSC participant enrollment by gender, aggregated across cohorts in FY20 (same time point as the interview collection). During FY20 approximately 64% of individuals were male and 35% female. Participants were 70% White, 28% Black/African American, 6% Multi-race, 1% Asian, less than 1% American Indian, and 34% Hispanic. See Table 2 for additional demographic details. Texas CSC diagnostic eligibility includes schizophrenia spectrum diagnoses, including affective psychosis. CSC participant diagnoses included: Schizophrenia (29%), Schizoaffective Disorder (25%), Bipolar Disorder with Psychosis (20%), Major Depression with Psychosis (20%), and other psychotic diagnoses (6%, e.g., Brief Psychotic Disorder, Unspecified Psychosis). Based on population statistics, 40% of the State of Texas identify as Hispanic and 13% identify as Black/African American which suggests an under-enrollment of Hispanic individuals throughout the state and an over-enrollment of individuals identifying as Black/African American.

**Table 2:** Client demographics for Fiscal year 2020 (Sept 2019-Aug 2020)

Site	#entering during FY	#exiting during FY	Average age	Percent Male	Race	Hispanic
#1 (*n* = 30)	*n* = 16, 53.3%	*n* = 11, 36.7%	23.8 (4.3)	*n* = 19, 63.3%	Black = 9, 30.0% White = 21, 70.0%	*n* = 5, 16.7%
#2 (*n* = 118)	*n* = 40, 33.9%	*n* = 50, 42.4%	20.5 (4.2)	n = 76, 64.4%	Asian = 1, 0.9% Black = 33, 28.0% American Indian = 1, 0.9% Multi-race = 14, 11.9% White = 69, 58.5%	*n* = 39, 33.1%
#3 (*n* = 18)	*n* = 18, 100%	*n* = 4, 22.2%	18.3 (3.8)	*n* = 8, 44.4%	White = 18, 100%	*n* = 18, 100%
#4 (*n* = 43)	*n* = 19, 44.2%	*n* = 22, 51.2%	20.2 (4.1)	*n* = 24, 55.8%	Black = 15, 34.9% White, 28, 65.1%	*n* = 5, 11.6%
#5 (*n* = 57)	*n* = 28, 49.1%	*n* = 28, 49.1%	21.1 (3.4)	*n* = 37, 64.9%	Asian, 1, 1.8% Black, 10, 17.5% Multi-race, 2, 3.5% White, 44, 77.2%	*n* = 2, 3.5%
#6 (*n* = 27)	*n* = 24, 88.9%	*n* = 10, 37.0%	23.8 (5.0)	*n* = 21, 77.8%	Black, 9, 33,3% Multi-race, 4, 14.8% White, 14, 51.9%	*n* = 7, 25.9%
#7 (*n* = 35)	*n* = 19, 54.3%	*n* = 18, 51.4%	22.9 (4.1)	*n* = 23, 65.7%	Black = 1, 2.9% Multi-race = 1, 2.9% White = 32, 94.3%	*n* = 19, 54.3%
#8 (*n* = 27)	*n* = 23, 85.2%	*n* = 8, 29.6%	22.1 (3.2)	*n* = 14, 51.9%	Black = 9, 33.3% Multi-race = 1, 3.7% White = 17, 63.0%	*n* = 2, 7.4%
#9 (*n* = 30)	*n* = 19, 63.3%	*n* = 8, 26.7%	21.7 (3.7)	*n* = 19,63.3%	Black = 13,43.3% Multi-race = 2, 6.7% White = 15, 50.0%	*n* = 2, 6.7%
#10 (*n* = 50)	*n* = 19, 38.0%	*n* = 23, 46.0%	19.9 (4.2)	*n* = 34, 68.0%	Black, 1, 2.0% White, 49, 98.0%	*n* = 29, 58.0%
#11 (*n* = 52)	*n* = 19, 36.5%	*n* = 18, 34.6%	21.2 (4.1)	*n* = 42, 80.8%	White, 34, 65.4% Black, 11, 21,2% American Indian, 1, 1.9% Multi-race, 4, 7.7% Asian, 2, 3.9%	*n* = 1, 1.9%
#12 (*n* = 35)	*n* = 28, 80.0%	*n* = 4, 11.4%	22.2 (4.0)	*n* = 22, 62.9%	Asian = 3, 8.6% Black = 17, 48.6% American Indian = 2, 5.7% Multi-race = 2, 5.7% White = 11, 31.4%	*n* = 5, 14.3%
#13 (*n* = 105)	*n* = 46, 43.8%	*n* = 36, 34.3%	22.4 (3.4)	*n* = 68,64.8%	Black = 57, 54.3% Multi-race, 4, 3.8% Pacific Islander = 1, 1.0% White = 43, 41.0%	*n* = 29, 27.6%
#14 (*n* = 31)	*n* = 10, 32.3%	*n* = 11, 35.5%	19.5 (3.8)	*n* = 19, 61.3%	Asian = 2, 6.5% Black = 12, 38.7% Multi-race = 2, 6.5% White = 15, 48.4%	*n* = 8, 25.8%
#15 (*n* = 21)	*n* = 5, 23.8%	*n* = 7, 33.3%	21.1 (4.3)	*n* = 10, 47.6%	White = 21, 100%	*n* = 1, 4.8%
#16 (*n* = 12)	*n* = 9, 75.0%	*n* = 4, 33.3%	20.8 (3.8)	*n* = 6, 50.0%	Asian = 1, 8.3% Black, 5, 41.7% White, 6, 50.0%	*n* = 1, 8.3%
#17 (*n* = = 28)	*n* = 26, 92.9%	*n* = 2, 7.1%	22.5 (3.9)	*n* = 16, 57.1%	Asian = 2, 7.1% Black = 9, 32.1% American Indian = 1, 3.6% Multi-race = 5, 17.9% White = 11,3 9.3%	*n* = 7, 25.0%
#18 (*n* = 45)	*n* = 22, 48.9%	*n* = 21, 46.7%	20.9 (3.9)	*n* = 27, 60.0%	Black = 6, 13.3% Multi-race = 8, 17.8% White, 31, 68.9%	*n* = 17, 37.8%
#19 (*n* = 31)	*n* = 27, 87.1%	*n* = 6, 19.4%	20.9 (4.4)	*n* = 15, 48.4%	Black, 2,6.5% Multi-race, 2, 6.5% White = 27, 87.1%	*n* = 9, 29.0%
#20 (*n* = 110)	*n* = 38, 34.6%	*n* = 48, 43.6%	21.7 (3.9)	N = 70, 63.6%	Asian = 1, 0.9% Black = 44, 40.0% Multi-race = 2, 1.8% White = 63, 57.3%	*n* = 48, 43.6%
#21 (*n* = 18)	*n* = 13, 72.2	*n* = 3, 16.7%	22.2 (3.5)	*n* = 10, 55.6%	Asian = 1, 5.6% Black = 1, 5.6% Multi-race = 1, 15.6% White = 15, 83.3%	*n* = 4, 22.2%
#22 (*n* = 58)	*n* = 29, 50.0%	*n* = 23, 39.7%	20.9 (3.8)	*n* = 34, 58.6%	White = 58, 100%	*n* = 57, 98.3%
#23 (*n* = 10)	*n* = 10, 100%	*n* = 3, 30.0%	21.1 (3.7)	*n* = 6, 60%	Black = 1, 10.0% Multi-race = 1, 10.0% White = 8, 80.0%	*n* = 0, 0%

### CSC Team Composition and Structure

CSC site Team Lead interviews revealed that team composition was fairly consistent across CSC sites. Differences were largely attributed to unfilled positions related to workforce shortages and site rurality. The OnTrackNY model outlines the following CSC team roles: 1) *Team Lead;* 2) *Primary Clinician;* 3) *Outreach Specialist;* 4) *Supported Employment/Supported Education Specialist (SEES);* 5) *Prescriber (e.g., Psychiatrist, Psychiatric Advanced Practice Nurse, or Physician Assistant; 6) Team Nurse; 7) Peer Specialist.* According to the OnTrackNY manual (Bennett, 2018), the Team Lead, Primary Clinician and Outreach Specialist can be combined into one role. Texas HHSC contracts require each CSC team to include at least a/an: (1) *Team Lead/Primary Clinician/Outreach Specialist* who is a licensed mental health provider in the State of Texas; (2) *Supported Employment/Supported Education Specialist (SEES);* (3) *Skills Trainer/Qualified Mental Health Provider;* and 4) Psychiatric *Prescriber (e.g., Psychiatrist, Psychiatric Advanced Practice Nurse, or Physician Assistant.* Additionally, sites should have access to a *Certified Peer Specialist* and *Certified Family Partner* if a full-time Peer or Family Partner is not assigned to the team (Bennett, 2018). In Texas, Peer Support Specialists and Family Partners complete state certification training (Texas HHS, 2022); sites cover training certification costs if individuals are not certified at the time of hire. Due to the immense mental health staff shortages statewide, the contract states programs can combine any CSC roles, or share with other programs if approved by the state mental health authority.

There are three primary differences between OnTrackNY model roles and State of Texas interpretations. First, the State of Texas includes the role of a *Skills Trainer/Qualified Mental Health Provider* which is a bachelor’s level role within the State of Texas akin to the community support provider role for adults with serious mental illness first developed in 1977 (Dixon & Goldman, 2003; Tessler & Goldman, 1982; Turner & TenHoor, 1978). However, there is no definition or outline of duties for the *Skills Trainer/Qualified Mental Health Provider* role making it unclear what elements of the traditional *Primary Clinician* roles they should fulfill. Second, as described above, the Texas state contract includes a certified family partner (CFP) role, as a new role. The Certified Family Partner provides non-clinical family-to-family support to CSC participants’ family members, for children and adults who consent to family involvement. Finally, the State of Texas does not mandate a *Nurse* as a part of the team. Almost universally, community mental health providers in Texas have nurses to support *Psychiatric Prescribers*. CSC Team Lead interviews suggest that nurses were leveraged for medication management and monitoring vital signs and did not have formal roles in CSC delivery.

Approximately half of the CSC sites had a combined *Team Lead and Primary Clinician* role (n = 12); while the other half separated the *Team Lead* and *Primary Clinician* role into two full-time positions (n = 11). Several sites with combined *Team Lead/Primary Clinician* roles described *“delegating up”* CSC-related administrative duties (e.g., data reporting) to the CSC *Team Lead’s* Supervisor (e.g., *Program Manager/Director*) to allow for greater focus of the *Team lead/Primary Clinician* on CSC clinical service coordination and delivery. Most sites (n = 18) had a *Supported Employment and Education Specialist (SEES)* position; however, five were in the process of filling this position at the time of data collection. Two sites reported that the *SEES* role was combined with the *Skills Trainer* role. Most sites had a *Skills Trainer* role (n = 21) but used various titles for the role, e.g., *Case Manager, Recovery Coach, Qualified Mental Health Professional (QMHP; i.e., defined as a person with a bachelor’s degree and at least two years of relevant experience)*. Sites without a *Skills Trainer* were new and attributed the lack of the role to their current small caseload size.

Fourteen CSC sites had a *Family Partner.* Nine had a fully designated *Family Partner*, while five shared a *Family Partner* with one or more programs in the same agency. Sixteen CSC sites had a *Peer Specialist*, of whom seven were part-time and/or shared with other programs in the same agency. Most CSC sites partnered with a specific internal *Prescriber* assigned to their team; however, four teams had a different structure. One site without an assigned internal prescriber connected CSC participants to any agency-employed adult prescriber, another site trained two psychiatrists in the model to serve CSC participants, and the last site allowed CSC participants to work with their CSC prescriber or to choose to see any other agency prescriber if they had pre-existing relationship. Finally, one site partnered with a local university medical school, and a fourth-year medical school resident was assigned to their site each year as the team *Prescriber.* See Table 3 for comparison.

**Table 3:** Comparison between OnTrackNY and Texas CSC Contract

Roles	OnTrackNY	Texas contract
Team leader	Required, full-time role	Required and roles are combined in the Contract Contract states they must be a Licensed Mental health provider Full-time role
Primary clinician	Required (can be combined with Team Lead)	
Outreach and recruitment specialist	Required (can be combined with Team Lead)	
Prescriber	Required, part-time role	Required, part-time role
Individual supported employment/supported education specialist	Required, full-time role	Required, full-time role
Nurse	Required, part-time role	Not part of State of Texas model
Peer specialist	Required	Required, can be part-time and must be certified through State Peer Specialist training
Certified family partner		Was not required at the time of data collection, added as a requirement in more recent contracts. Can be part-time
Skills trainer		Required, Full-time. Must standard to be a qualified mental health specialist in the State of Texas

### Team Lead Perspectives on CSC Implementation Challenges

#### Staff Training, Development, and Supervision

All Team Leads described the challenge of ensuring CSC staff were effectively trained in the OnTrackNY model and philosophy. Team Leads expressed concern that newer CSC staff did not receive the intensity or depth of training as compared to staff who were part of initial implementation efforts. Team Leads were overwhelmingly positive about regular peer-to-peer calls facilitated by the state authority and the regional SAMHSA technical assistance provider. CSC peer-to-peer calls began with Cohort 1 and expanded over time. When new sites were onboarded, they participated in monthly, one-on-one calls with state authority personnel and cohort specific calls. Over time, the newer cohorts were added to calls with Cohort 1 to provide site peer-to-peer support. The regional MHTTC technical assistance calls were open to CSC providers across a five-state region in the south southwest. Three different calls occurred: a general CSC team call, a peer and family partner learning call, and a learning collaborative focused on Supported Employment and Education best practices. CSC sites expressed a need for on-going, Texas-based OnTrackNY annual booster training to support new sites and staff turnover—“*I do wish that there would just be more of a state coordinated training every year that we can just do refreshers on with the team.”* Several sites expressed needs for additional training, including: Individual Placement and Support Supported Employment and Education, Cognitive Behavioral Therapy for Psychosis, crisis intervention, and adolescent and young adult engagement.

#### CSC Community Outreach and Enrollment

According to CSC *Team Leads,* most CSC referrals came internally from other agency programs and externally from local psychiatric hospitals. Cohort 1 sites were established in very populous urban areas where multiple referral streams quickly developed supporting swift client enrollment. Cohort 1 had a number of community connections that generated referrals reliably. For example, a Cohort 1 Team Lead stated: “*At any given time, we're working with our psychiatric hospitals, they may identify it first there; we're working with ER's, and working with mental health deputies. We're working with our crisis teams. We're working with our school that we are involved in.”* Some sites also had *“a hospital liaison within [the agency] who helps coordinate referrals… They are stationed at hospitals and their job is to help link people in the hospital to [the agency].”* However, newer cohorts struggled substantially in July 2020 with outreach and enrollment, in part because many new sites were rural and covered large geographic regions that logistically made community outreach and education challenging. They were also challenged by establishing referral pathways during the COVID-19 pandemic. A *Cohort 3 Team Lead* remarked: “*We have really literally exhausted the internal resources and just, we cannot find people. Either they don't want to participate, qualify but they just say, ‘no.’ I mean, we can't find people until they are past the DUP [duration of untreated psychosis] point.”* Sites discussed talking through these challenges on their monthly CSC site peer-to-peer support calls (provided by the state authority) to brainstorm ways to expand outreach during the peak of the pandemic.

Challenges to implementing a community-based program across a large catchment area included barriers related to driving significant distances, lack of public transportation, and scheduling. Indeed, sites serving one large, urban county reported having to navigate seeing individuals at all ends of a major metropolitan area, whereas more rural sites have individuals who live hours apart from each other. Urban counties commented about driving across large metropolitan areas: *“We serve all of [name] county from one end to the next, sometimes in one day.”* Similar comments were made by rural teams who drove many miles through large, expansive counties. *“We get referrals far away.”* However, Team Leads noted that Medicaid billing changes during the pandemic allowed greater flexibility. One rural site noted: “*We've been providing phone services or video conference services* [mixed with in-person services]*, and with that we're able to fit more individuals into our schedule.”* Although travel was a barrier, Team Leads were committed to the community-based nature of CSC, and saw it as a necessity and asset. Notably, all sites reported services largely occurred in the community, and no sites were providing in-clinic services beyond medication management.

#### CSC Team Member Role Specification, Integration and Collaboration

Team leads noted role clarification and overlapping responsibilities were problematic when implementing new CSC sites. One Team Lead remarked: *“SEES doesn't do peer work, case manager doesn't do peer work. Peer doesn't do employment stuff. Because, initially there was a lot of crossover and it caused some confusion.”* During early implementation, CSC teams went through a process of clarifying each team member’s role to improve collaboration and productivity over time. Team Leads described how team meetings were important for ensuring clear role delineation. Texas HHSC requires CSC sites to meet as a team at least twice monthly, yet most sites reported meeting twice weekly. Some teams involve all members while, some sites do not include their psychiatrist, peer, or family partner since they do not work full time for the team. Team Leads described daily phone and text among CSC team members to support collaboration in meeting client and family needs: “*Well, we're always in constant communication. We have our staffings every Monday, so we sit as a group and go through the list of our clients and discuss what's going on with them. On Wednesdays, we staff with our prescriber, so she's available to us. We talk about the clients that are going to get seen that day.”*

Team Leads felt CSC’s aim to directly support vocational goal attainment was an engagement tool. A Team Lead stated: “*I think for our program, it draws people into our program. When we're explaining our services to, especially our 18 and older population, that's a part of our services that they're really excited about.”* However, successful integration of *SEES* role into CSC teams, and prioritizing CSC participant vocational goals, proved challenging. First, CSC sites reported conflicts and confusion around what is considered a productive use of time in engaging young people around and supporting their vocational development; and ultimately what activities are deemed “billable” or not. One Team Lead stated: *“You have to have billable services, otherwise X, Y, and Z. And a lot of what SEES does is just not billable.”* Five CSC sites did not have a designated SEES role largely because of hiring difficulty and combined roles while trying to fill the position. CSC sites where the SEES role was integrated into Case Management and/or Primary Clinician roles struggled with prioritizing vocational support with case management, skills training, outreach and clinical interventions – ultimately decreasing consistent access to vocational support: *“The consistency of trying to provide the support and employment services to each of our clients, can become an issue from time to time.”*

All Team Leads reported that they and their team members were new to the SEES role, despite that most agencies provided evidence-based Individual Placement and Support (IPS) Supported Employment services to adults. (IPS services typically were housed in agency adult Intellectual/Developmental Disability programming; Cohen et al., 2020a, 2020b). Team Leads described how Supported Employment at their agencies is different than their SEES services: “*One of the things I found in our agency was that Supported Employment was only for people in the action stages of change. So, I had to go and rehabilitate and redefine what supported employment looks like when people are in different stages of change. And so that was a barrier and a challenge.”* CSC participants present with varying and dynamic levels of motivation in regards to exploring and engaging in work and school – and the SEES role serves all young people on the team – not just those who are motivated and referred to the SEES. Team Leads expressed a desire for SEES training and additional support with SEES integration, which was the motivation for the subsequent MHTTC learning community. Only two CSC sites had supported their SEES in attending Individual Placement and Support (IPS) Supported Employment training. These sites described how they adapted IPS practices to better meet the needs of young people to better support career exploration and connection to pre-employment opportunities (e.g., volunteering) to build skills and confidence in order to be successful with competitive employment. One Team Lead stated:* “I would say that we've definitely made adaptations from the [IPS] model… And also, volunteer opportunities have not been historically thought of as a viable asset of Supported Education and Employment. But for young people who don't have much work experience and don't really have much sense of agency, volunteering can be a real safe way to develop occupational skills and social skills.”*

#### Unique Developmentally-Related Client Needs

Twenty of 22 Team Leads reported that their CSC team struggled with recruiting, engaging, and sustaining CSC participation among adolescents. One remarked: “*We haven't had any adolescents yet on our team. All of our clients right now are early twenties to mid-twenties.”* Most Team Leads reported having little to no clinical experience outside of programs for adults with serious mental illness. Many Team Leads expressed how engaging young people and families was different than middle-aged adults. A Team Lead stated: “*When we do get to the [adolescent and young adult] referrals, making sure that we can try to keep them engaged despite the education and the barriers and the family issues. I think there's a lot more that goes into it with an adolescents' enrollment.”* Team Leads expressed that that young people wanted flexibility to engage and reengage as they wanted or needed supports. And, although this work is challenging at times, many Team Leads expressed joy in developing young person-friendly, creative engagement strategies, and valued seeing progress made among their sites’ participants: *“They’re staying in school or they're getting their GED or they're going back to school or they're starting college classes. And I think that's something that is really great because a lot of them have told us, ‘I don't think I'd be able to do this without this help.’”.*

Further, most Team Leads described limited experience with family involvement in mental health service delivery. As such, many Team Leads expressed challenges with engaging families, supporting shared decision-making with young people and their families, and navigating conflict resolution between youth and families. One stated: “*We need to reorganize to be able to involve families from the very beginning and help them to encourage their participation and hope it'll improve the outcome of the services and the recovery. But definitely is it going to be a challenge.”* Most Team Leads spoke about learning to have conversations from participant intake around consent to involve at least one chosen family member in CSC services. One stated: “*We definitely start with making sure we get some level of consent so that we can reach out to them. We let the consumers know that if we reach out to family, we're going to inform them and that usually makes them a little bit more comfortable.”* Despite being new to serving families, Team Leads embraced the importance of including family in services because they described that young people may benefit from guidance, reinforcement and support to maintain their recovery, and also that CSC services are designed to be temporary:* “Once we [CSC team] leave, we need to know that somebody is going to be able to help them stay focused on their recovery and navigate it.”*

CSC sites with Certified *Family Partners* provided additional tailored support and education to families. Team Leads described *Family Partners* as critical in serving as a sounding board for validating and processing worry and frustrations as well as mitigating and diffusing family conflict. One stated: “*They [client family members] have more of an area to express their frustrations, especially with their Family Partner.”* Beyond individual family peer support services, a few CSC sites provided family groups. “*We started family support and it has been really good. We have families that come every single week.”* Some sites had family-only psychoeducation and/or support groups, while others had multi-family groups (McFarlane et al., 2015) that included young people and families together.

## Discussion

This is the first study aiming to understand the growth of CSC programs in Texas, demographics of individuals served, the CSC model in Texas (adapted from OnTrackNY), and perceived barriers to implementation. It integrates quantitative administrative data, content from contracts and qualitative interviews from the Team Lead’s perspective to provide a multidirectional understanding of CSC implementation. The scope of CSC implementation across the state of Texas is noteworthy. In five years, the state mental health authority directed federal MHBG funds to open CSC sites within 24 separate organizations with the ability to serve between 30 to 90 young people at each site. To date, the state has expanded to 29 of the 39 public mental health providers. Overall, lessons learned in Texas CSC implementation are relevant to and have implications for national CSC expansion efforts. These include the state’s successful adaptation of CSC, CSC site peer-to-peer learning, and integration of Case Managers, Peer Support specialists and Family Partners, as well as the need for CSC training adaption for local context/system and CSC team further developmental and cultural attunement to improve CSC client engagement and support strategies. There is a national need to better understand how states are adopting CSC and the impact of adaptations, as well as what types of state training infrastructure are needed to sustain CSC and associated adaptations.

Study findings suggest that state contracts, paired with peer-to-peer CSC site training and consultation, were effective in establishing CSC sites across rural, suburban and urban settings. Despite Texas not mandating specific CSC training (and the substantial rurality, geography, and demographic differences between sites), CSC sites were relatively similar in CSC model elements and practices. State contract language mirrored language found in key NIMH CSC dissemination documents (e.g., Heinssen et al., 2014; Bennett et al., 2018). Both state- and SAMHSA-sponsored site-to-site peer calls appeared to ensure relative CSC site uniformity in Texas. Pogue, et al. (2022) outline how *Learning Collaboratives* can reinforce evidence-based practice implementation across organizations. However, evidence-based practice *Learning Collaboratives* typically are paired with staff training and fidelity assessment to monitor adherence to an evidence-based practice model (Dixon & Patel, 2020). Texas CSC site *Team Leads* described a need for initial and on-going state-specific training to support implementation. While the Texas CSC programs relied on experienced training organizations to get established, they are now desiring more formal in-state infrastructure to support and sustain CSC. The regional MHTTC has integrated additional virtual trainings based on CSC peer learning collaborative identified training needs, including training in Cognitive Behavioral Therapy for psychosis, and Young Adult Individual Placement and Support Supported Employment and Education.

### Unique Texas CSC Features

This study suggests that full-time *Skills Trainer/QMHP, Family Partner* and *Peer Support Specialists* can be successfully integrated into CSC. With high workforce shortage rates, the Texas public mental health system relies on the bachelor’s level *Skills Trainer/QMHP* role throughout the system. The NAVIGATE CSC Model has a practice called *Individual Resiliency Training* designed to be provided by a master’s level mental health professional (Meyer et al., 2015). Most Texas CSC sites had a *Skills Trainer/QMHP* role as a stand-alone role on the CSC team. However, the Texas CSC contract did not explicitly describe the *Skills Trainer/QMHP* duties. An international CSC fidelity scale explicitly states that each client must be *“assigned a case manager or care coordinator”* (Addington, 2021), yet CSC coordination and case management philosophies and processes are not well-defined. Most CSC case management and care coordination is conducted by primary clinicians, who are often licensed clinical social workers, counselors, and psychologists. Further, case management and care coordination vary across CSC models. There is opportunity in Texas to further examine how/whether a non-licensed bachelors-level *Skills Trainer/QMHP* role: (1) is integrated into the CSC team, (2) partners with team members, (3) uniquely supports young people, and (4) impacts cost/cost-savings and outcomes.

At the time of data collection, many Texas CSC sites had both full-time *Peer Support Specialists* and *Family Partners*. Since the Team Lead interview data collection, the state contract has evolved to require *Family Partner* role in addition to the *Peer Support Specialist* role at least part-time on all CSC teams. However, *Family Partners* and *Peer Support Specialists* are not included in the international CSC fidelity scale (Addington, 2021). To our knowledge, there are not any CSC models that have formally adopted *Family Partners.* And, although *Peer Support Specialists* are increasingly being integrated into CSC teams (SAMHSA, 2019), few CSC Models formally include *Peer Support Specialists.* The notable exception is OnTrackNY (see Peer Support Manual, DuBrul et al., 2017). *Peer Support Specialists* are trained and certified mental health professionals who use their lived experience with navigating life and mental health services successfully with a mental health diagnosis to validate client experiences, instill hope, provide for a sense of belonging and that one is not alone, advocate with and for, and provide non-stigmatizing tailored support. OnTrackNY views *Peer Support* as critical for providing individual support, but also for ensuring that CSC team operations are recovery-oriented and developmentally-attuned (DuBrul et al., 2017). The evidence base for CSC Peer Support is growing (Hopkins et al., 2020; Nguyen et al., 2022; White et al., 2017). Given the state mandated adoption of Peer Supports across the state through the CSC contract, Texas is poised as a location to study the impacts of *Peer Support Specialists* on engagement and recovery domains, mechanisms for this impact, and how they uniquely contribute to CSC team operations, developmental-attunement, and recovery-orientation.

Family involvement and psychoeducation are core features of CSC models and linked to treatment engagement and mental health symptom reduction (Lucksted et al., 2015; McFarlane et al., 2015). However, inclusion of a *Family Partner*, a “secondary peer” role is rare. *Family Partners* have lived experience as caregivers of a young people diagnosed with mental health conditions. *Family Partners* are trusted guides with helpful information and resources, role models for communication, advocacy and self-care, supports in the navigation of complex systems (e.g., mental health, education, legal), and provide emotional support and connection. The *Family Partner* model originated through the child mental health SAMHSA System of Care movement. Research suggests that families who participate in *Family Partner* services have more knowledge about symptoms, reduced stress (Jamison et al., 2017), less parental anxiety, higher satisfaction with care, higher participation in services, and better social connectedness than those receiving care as usual (Radigan et al., 2014; Bearman, et al., 2022). Texas provides the opportunity to study how the *Family Partner* role uniquely impacts young person and family service engagement, in particular if the role increases trust between CSC teams and families of historically minoritized populations.

### Need for Youth Developmental Expertise & Cultural Attunement

Team leads reported challenges in engaging adolescents, which was affirmed by low rates of 14–17-year-old CSC participation observed in administrative data. All 23 Texas CSC sites were located in adult community mental health service settings with adult community mental health leadership. Team Leads largely reported both their and their CSC team members’ past work experience was in adult mental health settings. Based on the structure of mental healthcare in which most providers segment themselves into child or adult providers, it is rare to find providers with both adolescent and young adult expertise. Although child providers are more likely to have experience navigating adolescent development, family relationships, and school systems important to CSC care, they are less likely than adult providers to have expertise in psychosis. As such, it may be challenging for CSC programs to find providers that have experience with both psychosis and child development (Klodnick et al., 2021). However, CSC dissemination across the US is novel in focusing on the transition-to-adult populations when psychosis onset is most prevalent (Comacchio et al., 2019; Kessler et al., 2007). Texas CSC implementation highlights how challenging it can be for adult provider workforce, programs, and systems to engage transition-aged youth – even with inclusion of *Peer Support Specialists* and *Family Partners.* Texas CSC *Team Leads* noted the struggles of working across child and adult agency and state policies, in addition to, learning how to navigate typical adolescent behaviors and family conflict. NIMH CSC materials suggest that CSC is derived from adult community mental health evidence-based practices (e.g., Assertive Community Treatment, IPS Supported Employment; Heinssen et al., 2014). There is real opportunity to integrate youth-focused interventions and developmental considerations, such as Positive Youth Development, as well as youth-focused principles and practices (Catalano et al., 2004; Klodnick et al., 2022; Lerner et al., 2009; Shek et al., 2019) into CSC programs, and to examine the impact that having more developmentally-attuned CSC teams have on adolescent engagement and outcomes.

Administrative data revealed high representation of Hispanic and Black participants, speaking to the importance of culturally-responsive care. This over-representation of Black individuals has been found across other programs in the US. Daley et. al (2022) recently found that 70% of a sample of 35 CSC programs served a disproportionately higher rate of Black individuals as compared to the local service area. These findings raise important questions for both outreach protocols and culturally responsive care. Although Team Leads did not discuss race or culture in regards to outreach or cultural competency more broadly as a barrier, cultural competence/humility has previously been identified as a barrier to CSC implementation (Powell et al., 2021). A growing literature documents race and ethnicity-related disparities in early psychosis program outcomes (Oluwoye et. al, 2018, 2020, 2021). Understanding mechanisms that reproduce such disparities in Texas is critical for effective implementation, given the substantial diversity in Texas cultures, races, ethnicities, and languages. Approximately 40% of Texans self-identify as Hispanic/Latino, 13% Black/African American, 5% Asian, and 42% White, Non-Hispanic. Per the 2009–2013 US Census Bureau, 65% of individuals over age five speak only English at-home. Over 160 languages are spoken in Texas homes, with 85% speaking Spanish and other languages, including Vietnamese, Chinese, Tagalog, German, French, Hindi, Urdu, Korean, and Arabic (US Census Bureau, 2020). As CSC sites continue to grow in Texas, it is critical to better understand and address factors related to or (re)producing disparities, including workforce diversity, culture and linguistic needs training, and systems-level transformation.

The authors of this paper recently received funding to be a part of the national Early Psychosis Intervention Network (EPINET) National Institute of Mental Health (NIMH)-funded project, which will present opportunities for collaboration and evaluation across states. Future research plans include examination of rural versus urban team models, structures, and outcomes, which is critical to effective implementation across Texas. The EPINET-TX project is developing data harmonization infrastructure across 15 CSC sites in Texas, participatory research methods, and principles of a learning healthcare system that will present opportunities for better understanding CSC outcomes, disparities, and needed quality improvement initiatives.

## Limitations

This study provides specific results about one US state. The results may help other US states earlier in implementation to foresee potential barriers; however, barriers may be unlikely to transfer to an international context. Although the results include Team Lead perspectives, contracts, and administrative data across 23 CSC sites, no fidelity measure was used to establish if the sites were in fact implementing CSC in accordance with one or more models. Interviews were only conducted with CSC site Team Leads, and did not include other site CSC team members, state administrators, OnTrackNY trainers, youth or families. Additional perspectives may have revealed additional CSC implementation challenges and solutions. *Team Leads’* interview data was also collected virtually at the beginning of the COVID-19 pandemic when sites were having substantial issues with workforce turnover, and young person and family engagement via technology.

## Conclusion

This study uses a mixed-methods cross-sectional approach to describe CSC implementation efforts and barriers in Texas. Findings not only have significant implications for informing national CSC expansion efforts, but also for implementation of other evidence-informed mental health treatment models that target youth and young adults. Future research must examine correlates and predictors of implementation challenges and their relation to client and family engagement and outcomes. As implementation occurs more rapidly across the US, attention must turn to the research-to-practice and practice-to-research pipelines that can coordinate knowledge and collaboration across researchers, providers, individuals in services, and policy makers to increase effective implementation. EPINET-TX is using this study’s findings to shape trainings and technical assistance to CSC sites.
